# Tubular Assembly Formation Induced by Leucine Alignment along the Hydrophobic Helix of Amphiphilic Polypeptides

**DOI:** 10.3390/ijms222112075

**Published:** 2021-11-08

**Authors:** Mohammed A. Abosheasha, Toru Itagaki, Yoshihiro Ito, Motoki Ueda

**Affiliations:** 1 RIKEN Cluster for Pioneering Research (CPR), Wako 351-0198, Saitama, Japan; mohammed.abosheasha@riken.jp (M.A.A.); toru.itagaki@riken.jp (T.I.); y-ito@riken.jp (Y.I.); 2 Department of Biological Sciences, Graduate School of Science, Tokyo Metropolitan University, Hachioji 192-0397, Tokyo, Japan; 3 RIKEN Center for Emergent Matter Science (CEMS), Wako 351-0198, Saitama, Japan

**Keywords:** helix–helix interaction, helix orientation, secondary structure, peptide assembly

## Abstract

The introduction of α-helical structure with a specific helix–helix interaction into an amphipathic molecule enables the determination of the molecular packing in the assembly and the morphological control of peptide assemblies. We previously reported that the amphiphilic polypeptide SL12 with a polysarcosine (PSar) hydrophilic chain and hydrophobic α-helix (l-Leu-Aib)_6_ involving the LxxxLxxxL sequence, which induces homo-dimerization due to the concave–convex interaction, formed a nanotube with a uniform 80 nm diameter. In this study, we investigated the importance of the LxxxLxxxL sequence for tube formation by comparing amphiphilic polypeptide SL4A4L4 with hydrophobic α-helix (l-Leu-Aib)_2_-(l-Ala-Aib)_2_-(l-Leu-Aib)_2_ and SL12. SL4A4L4 formed spherical vesicles and micelles. The effect of the LxxxLxxxL sequence elongation on tube formation was demonstrated by studying assemblies of PSar-*b*-(l-Ala-Aib)-(l-Leu-Aib)_6_-(l-Ala-Aib) (SA2L12A2) and PSar-b-(l-Leu-Aib)_8_ (SL16). SA2L12A2 formed nanotubes with a uniform 123 nm diameter, while SL16 assembled into vesicles. These results showed that LxxxLxxxL is a necessary and sufficient sequence for the self-assembly of nanotubes. Furthermore, we fabricated a double-layer nanotube by combining two kinds of nanotubes with 80 and 120 nm diameters—SL12 and SA2L12A2. When SA2L12A2 self-assembled in SL12 nanotube dispersion, SA2L12A2 initially formed a rolled sheet, the sheet then wrapped the SL12 nanotube, and a double-layer nanotube was obtained.

## 1. Introduction

In nature, proteins have highly ordered morphologies called tertiary or quaternary structures that are well-defined by precisely controlled packing of secondary structures via various noncovalent interactions such as hydrogen bonding, hydrophobic interactions, van der Waals interactions, electrostatic interactions, π–π interactions, and π–cation interactions [1]. Introducing a well-designed secondary structure with a specific interaction into an amphipathic molecule results in the design of an artificial peptide assembly that is precisely morphologically controlled. Woolfson and coworkers succeeded in designing a spherical structure in which helices were regularly arranged using a coiled-coil formation, which is a typical interaction between α-helices [2,3,4]. Conticello and coworkers prepared artificial nanotubes with uniform diameters by rational design of the orientation, inclination, and packing of the α-helices using the spatial arrangement of amino acids on the helix [5,6]. Additionally, his group reported that the helix–helix interaction known as the collagen triple helix is also available as a unit for the design of uniformly sized square nanosheets [7,8]. Such a combination of self-assembly of amphiphiles and helix packing controlled by a helix–helix interaction is a promising tool for the fabrication of soft matter with de novo designed morphology and well-defined molecule packing.

The concave–convex interaction between helices is an appropriate tool for determining helix packing because it can be used to rationally design the orientation and inclination of a helix in the helix packing based on structural effects only without any other adhesion force, like “LEGO” bricks. The concave–convex interaction is determined by the spatial positioning of amino acid side chains around the helix. As the concave–convex nature of the helix results from the bulkiness of the side chain, it has been reported that the leucine (Leu) position is very important. To date, the effective peptide sequences in a concave–convex interaction of α-helices have been determined from the structural study of dimerization between natural proteins. One such sequence is a heptad repeat sequence LxxLxxx that induced the coiled coil, where L is Leu and x denotes any other amino acid. This heptad repeat sequence is known to cause a tilted helix–helix interaction with a specific angle of 20° due to the concave–convex interaction known as “knobs-into-holes” packing [9,10,11]. Moreover, the helix of repeated 11-residue LxxLxxxLxxx also forms knobs-into-holes packing [12,13]. Furthermore, it was reported that the non-repeated sequences LxxxGxxxGxxxL [14,15] and LxxxLxxxL [16,17,18,19] also form a dimer with a specific tilt angle between helices and different packing from the knobs-into-holes arrangement. The surface irregularity created by a spatial arrangement of Leu side chains is therefore a key factor in determining a specific helix–helix interaction. These sequences are available for designing the orientation and axial tilt of neighboring helices in a peptide assembly of amphiphilic polypeptides with a hydrophobic helix.

We previously reported that nanotubes with a uniform diameter of ca. 80 nm were prepared from an amphiphilic block copolypeptide, PSar_n_-*b*-(l-Leu-Aib)_6_ (SL12) with a hydrophobic α-helix composed of the LxLxLxLxLxLx sequence, which involves two LxxxLxxxL sequences [20,21]. The phenomenon was explained by the hypothesis that the helical part is uniformly packed in a hydrophobic layer due to the concave–convex interaction between helices with the LxxxLxxxL sequence [20,21,22,23]. Kimura and coworkers demonstrated that PSar_n_-*b*-(l-Val-Aib)_6_ did not form a nanotube [24], which showed the importance of the Leu side chain for morphology control. Thus, although soft matter may spontaneously form from amphipathic molecules, its homogeneity and shape design are possible with the precise molecular orientation found in proteins. While it has been shown that SL12 assumes tube-forming packing, it has been reported that PSar_n_-*b*-(l-Leu-Aib)_8_ (SL16) forms vesicle structures [23,25,26]. Hence, in this study, we investigated the importance of the LxxxLxxxL sequence of the hydrophobic helix for tubular assembly formation by comparing self-assembly prepared from four kinds of amphiphilic polypeptides, SL12, SL16, PSar-*b*-(l-Leu-Aib)_2_-(l-Ala-Aib)_2_-(l-Leu-Aib)_2_ (SL4A4L4), and PSar-*b*-(l-Ala-Aib)-(l-Leu-Aib)_6_-(l-Ala-Aib) (SA2L12A2).

## 2. Results and Discussion

### 2.1. Amphiphilic Polypeptides

**Figure 1:**
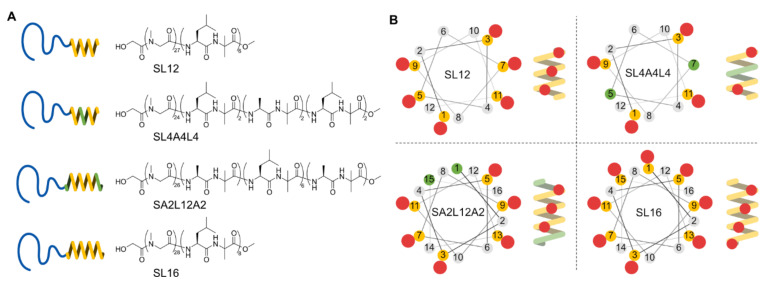
Schematic illustrations of chemical structures (**A**) and helical wheels (**B**) of amphiphilic polypeptides; SL12, SL4A4L4, SA2L12A2, and SL16. Yellow, green, and gray dots of helical wheels reflect the position of Leu, Ala, and Aib, respectively. Red dots indicate the positions of Leu side chains on the helix (**B**).

We designed PSar-*b*-(l-Leu-Aib)_2_-(l-Ala-Aib)_2_-(l-Leu-Aib)_2_ (SL4A4L4) to elucidate the importance of the LxxxLxxxL sequence for tube formation. Furthermore, we synthesized PSar-*b*-(l-Ala-Aib)-(l-Leu-Aib)_6_-(l-Ala-Aib) (SA2L12A2) to determine the effect of the concave–convex structure around both the *N*- and *C*-terminals of a 16 mer α-helix, on the tube-forming helix packing by comparing it with SL16 (Figure 1A).

**Figure 2:**
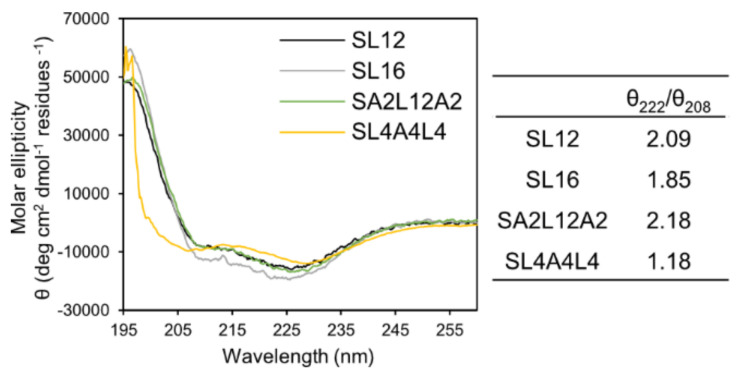
CD spectra and the ratios of θ_222_/θ_208_ of peptide assemblies prepared from SL12, SL4A4L4, SA2L12A2, and SL16 in milli-Q water.

The polysarcosine chain length 27, 24, 26, and 28 mers of the synthesized amphiphiles SL12, SL4A4L4, SA2L12A2, and SL16 were determined by MALDI-TOF MS and ^1^H NMR spectroscopy, respectively (Figures S1–S4). These hydrophilic chain lengths can be treated as the same. The length of 20–30 mers was chosen according to previous work [21,25,27]. Next, the secondary structure of the hydrophobic block of the amphiphiles was investigated by CD spectroscopy. All hydrophobic blocks of the peptides in the assemblies formed an α-helix structure and their helix contents per residue were not significantly different in milli-Q water (Figure 2). The ratios of *θ*_222_/*θ*_208_ for all samples were higher than 1, indicating that helices were densely packed to form a bundle in a hydrophobic layer of peptide assembly [28,29,30,31].

### 2.2. Tube Formation Induced by LxLxLxLxLxLx

**Figure 3:**
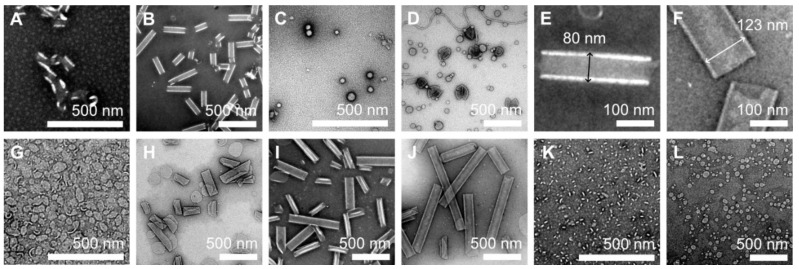
Negatively stained TEM images (samarium acetate stain) of peptide assemblies prepared from SL12 (**A**,**B**,**E**), SL4A4L4 (**C**,**D**), SA2L12A2 (**F**–**J**), and SL16 (**K**,**L**) after heat treatment at 90 °C for 0 h (**A**,**C**,**G**,**K**), 1 h (**B**,**D**,**H**,**L**), 3 h (**I**), and 6 h (**J**). (**E**,**F**) are the magnifications of (**B**,**J**). Scale bars are 500 nm (**A**–**D**,**G**–**L**) and 100 nm (**E**,**F**).

The ethanol injection method initially formed curved sheets of SL12 in milli-Q water. After heat treatment at 90 °C for 1 h, the sheets spontaneously transformed into uniform tubular structures with a diameter of around 80 nm (Figure 3A,B,E), which corresponds to previous results [21,22,23,25,27,32,33]. In contrast, a spherical micelle and vesicle were observed in the dispersion of SL4A4L4, which had the same length of a 12 mer hydrophobic α-helical block as SL12 (Figure 3C,D).

**Figure 4:**
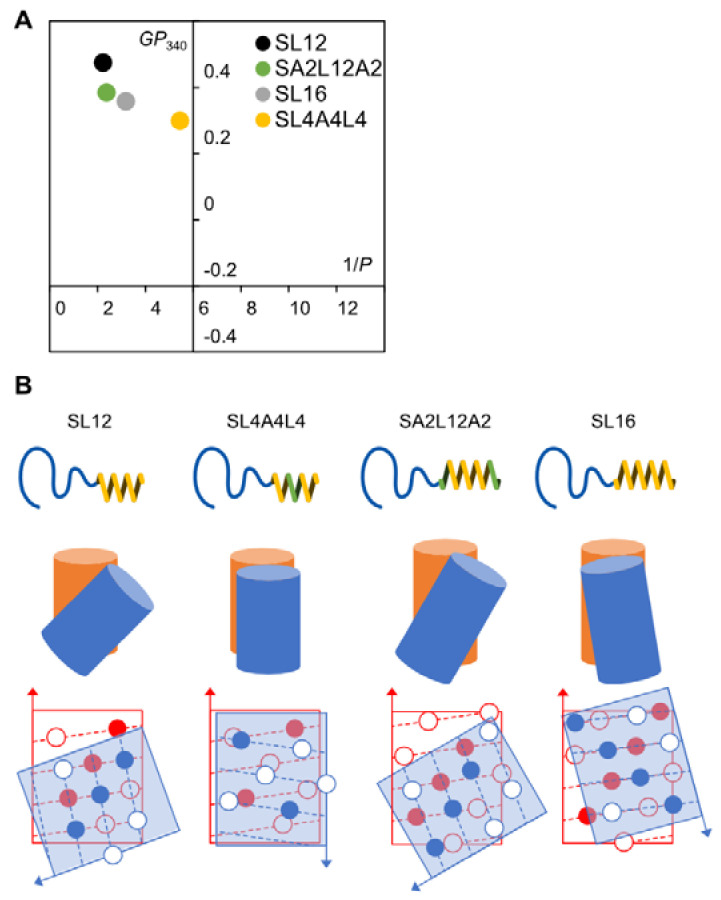
Cartesian diagram (**A**) and schematic illustration of the helix packing models (**B**) in peptide assemblies prepared from SL12, SL4A4L4, SA2L12A2, and SL16. The arrows in the helical net diagrams indicate the orientation from the *C*- to *N*-terminal. In the packing model (**B**), the orange and blue cylinders represent hydrophobic α-helical blocks of amphiphilic polypeptide and their neighbors, respectively.

This result indicates that the alignments of Leu1-Leu5-Leu9 and Leu3-Leu7-Leu11 along the axis of the helix (Figure 1B) are a key factor for the tubular formation. SL12 has a hydrophobic block of LxLxLxLxLxLx composed of two LxxxLxxxL sequences, which induces tilted helix packing known as a “ridges-into-grooves” model [18,19]. SL12 has two Leu-aligned sides (Leu1-Leu5-Leu9 and Leu3-Leu7-Leu11) and two Aib-aligned sides (Aib2-Aib6-Aib10 and Aib4-Aib8-Aib12), which form ridges-into-grooves packing and have no concave–convex interaction, respectively. The faces of Leu1-Leu5-Leu9 and Leu3-Leu7-Leu11 are at almost opposite positions around the helix because the angle between Leu1 and Leu3 is 200 degrees. Thus, SL12 is packed on the ridges-into-grooves basis with a tilted angle in a specific direction (Figure 4B). This induced anisotropy of peptide packing in the hydrophobic layer to enable an anisotropic structure like a tube. In contrast, SL4A4L4, which has a hydrophobic helix of LxLxAxAxLxLx, could not pack on the ridges-into-grooves basis owing to the lack of the middle Leu of LxxxLxxxL, resulting in it forming an isotropic spherical shape (Figure 4B).

Next, we checked the morphology of the SA2L12A2 and SL16 assemblies. SA2L12A2 initially formed a curved sheet like SL12. The sheet rolled up into multilayer rolled sheets after heating at 90 °C for 1 h and finally transformed into nanotubes after 6 h (Figure 3G–J). This result indicates that SA2L12A2 also induced the tilted helical packing derived from the ridges-into-grooves interaction to form nanotubes—like SL12—because SA2L12A2 has the tube-inducing sequence LxLxLxLxLxLx in the middle section of the hydrophobic block (Figure 4B). In addition, it was determined that the Ala residues of AxLxLxLxLxLxLxAx do not interrupt the ridges-into-grooves packing between the LxLxLxLxLxLx sequences of neighboring helices. Interestingly, SL16 formed a planer sheet and vesicles, as previously reported [25,26,34] (Figure 3K,L), even though SL16 also has the LxLxLxLxLxLx sequence. This spherical shape formation indicates that the hydrophobic block L16 induces different helix packing to L12. The Leu1 and Leu15 of SL16 clearly affected the helix packing because they also joined to make a ridge. A possible reason for the effect of Leu1 and Leu15 is that the angle between Leu1 and Leu13 of LxxxLxxxLxxxL is 120°. LxxxLxxxLxxxL may be too long to form ridges-into-grooves packing because a 120° angle between Leu1 and Leu13 or Leu3 and Leu15 is expected to require slight bending of the helix axis for packing like the coiled coil of the repeated heptad sequence, but the 16 mer helix may be too short and rigid to bend, resulting in the ridges-into-grooves packing not being thermodynamically stable.

Here, both SL12 and SA2L12A2 formed nanotubes, but their diameters were around 80 and 123 nm, respectively (Figure 3E,F). This difference comes from their different membrane curvatures, which are thought to be derived from the different tilt angles between the packed helices. The difference in tilt angle can be explained by the dipole–dipole interaction between helices. SA2L12A2 has a stronger dipole moment along the helix axis due to its longer α-helix structure than that of SL12 and, as a result, is expected to prefer more parallel helix packing than SL12 (Figure 4B). In the stable structure of the nanotube, SA2L12A2 showed a larger diameter, which means lower membrane curvature. In contrast, after short heat treatment, rolled sheets with very narrow diameter resulted for SA2L12A2. Thus, A2L12A2 formed tube-forming packing dominated by LxLxLxLxLxLx, and the Ala-Aib sequence might make the membrane flexible because the Ala-Aib sequence does not have a specific interaction with the neighboring Ala-Aib section to show a high degree of freedom of packing.

### 2.3. Membrane Fluidity

The membrane fluidity of each peptide assembly was investigated using fluidity-sensitive dyes, DPH and Laurdan, which can insert into the hydrophobic region and hydrophilic–hydrophobic interface of the amphiphile membrane, respectively [35,36,37]. We can estimate the helix packing from the membrane fluidity because the membrane fluidity directly reflects the rigidness of the molecular packing. The hydrophobic layer of SL12 and SA2L12A2 showed similar 1/*P* values of 2.23 and 2.37, respectively, but the hydrophilic–hydrophobic interface had a different fluidity, showing *GP*_340_ values of 0.474 and 0.384, respectively (Figure 4A). This result also supported our hypothesis that SA2L12A2 formed a similar helix–helix interaction in the LxLxLxLxLxLx region to that of SL12, but the hydrophilic–hydrophobic interface composed of Ala-Aib residues was slightly more fluid than that composed of Leu-Aib in the SL12 nanotube. In contrast, SL16 showed a higher 1/*P* value of 3.19 and lower *GP*_340_ value of 0.357 than SL12, which indicates a relatively fluid hydrophobic layer and interface compared with those of L12 (Figure 4A). This result also means that the SL16 assembly was formed by a different helix packing to the tube-forming packing derived from the LxLxLxLxLxLx sequence (Figure 4B). Finally, SL4A4L4 had significantly different 1/*P* and *GP*_340_ values of 5.45 and 0.299, respectively. Therefore, the membrane composed of SL4A4L4 might be fluid owing to the lack of a stable concave–convex interaction induced by the side chain of Leu in the middle of the helix.

### 2.4. Double Layer Nanotube Formation by SL12 and SA2L12A2 Nanotubes

**Figure 5:**
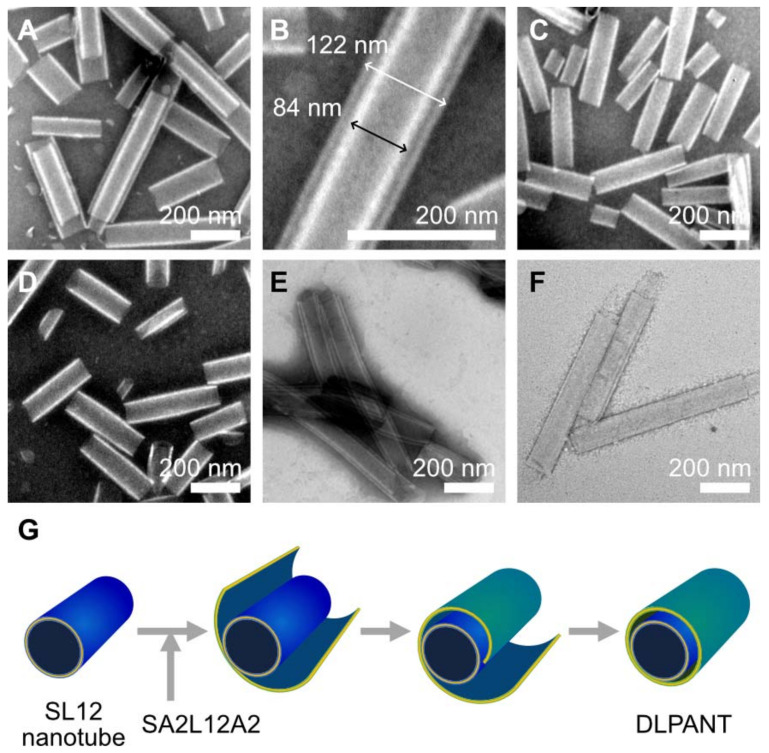
Negatively stained TEM images of assemblies prepared by SA2L12A2 self-assembling in a SL12 nanotube dispersion (**A**,**B**), SL12 self-assembling in a SA2L12A2 nanotube dispersion (**C**), and the self-assembly of a mixture of SL12 and SA2L12A2 (**D**). (**B**) is the magnification of (**A**). TEM observation of SA2L12A2 self-assembled on the surface SL12 nanotubes with 30% PEG (**E**) and subsequent purification to remove PEG through dialysis (**F**). Schematic illustration of the formation mechanism of DLPANT (**G**). Scale bars are 200 nm.

Precise design of the peptide sequence of the hydrophobic helix A2L12A2, which was composed of a tube-forming sequence of LxLxLxLxLxLx as a middle section and inert sequence of Ala-Aib as both terminals, enabled the fabrication of slightly thicker nanotubes compared with the nanotubes prepared from L12. When SA2L12A2 was allowed to self-assemble in a SL12 nanotube dispersion, a double-layer peptide amphiphile nanotube (DLPANT) was obtained as a minor fraction with a low yield of around 10%, which was calculated from TEM images (Figure 5A,B and Figure S5). In contrast, when we tried to self-assemble SL12 in a SA2L12A2 nanotube dispersion, no DLPANT was observed (Figure 5C). In addition, a mixture of a SL12 nanotube dispersion and SA2L12A2 nanotube dispersion—prepared separately—maintained the independent nanotube structures even after heat treatment at 90 °C (Figure 5D). These results are explained by the hypothesized mechanism that SA2L12A2 formed a sheet, and the sheet wrapped the SL12 nanotube to form a double-layer nanotube (Figure 5E). The inner and outer nanotube diameter of the double-layer nanotube were around 84 and 123 nm, which corresponds to each nanotube comprising a single component of SL12 or SA2L12A2 (Figure 5B).

To study the effect of temperature on DLPANT formation, a mixture of SL12 nanotubes and SA2L12A2 solution was heated at 50 and 70 °C for 6 h. In both cases, DLPANT was not obtained (Figure S6). This could be because the polysarcosine layers of the SA2L12A2 sheet and SL12 nanotube were dehydrated at 90 °C, and the SA2L12A2 sheet spontaneously wrapped the surface of the SL12 nanotube due to an enhanced hydrophobic–hydrophobic interaction. To further promote hydrophobic interaction among the assemblies to improve the yield of DLPANT formation, we added PEG solution to the SL12 nanotube dispersion (final concentration of PEG 30%), then injected SA2L12A2 ethanol solution into the mixture, followed by heat treatment at 90 °C for 3 h. PEG is known as a dehydration agent and has been reported to promote hydrophobic interaction and sticking between assemblies due to the dehydration of hydrophilic surfaces [38,39]. As a result, DLPANT, as the major fraction, as well as a multi-layer peptide amphiphile nanotube (MLPANT), were obtained with a higher yield of >95% (Figure 5E and Figure S7). After removal of the PEG via dialysis, DLPANT and MLPANT remained (Figure 5F).

## 3. Materials and Methods

### 3.1. Materials

All amino acids and condensation reagents were purchased from Watanabe Chemical Ind., Ltd., Hiroshima, Japan. Membrane fluidity sensitive dyes, 1-[6-(dimethylamino)-2-naphthalenyl]-1-dodecanone (Laurdan, CAS:74515-25-6, Cayman Chemical Company, Ann Arbor, MI, USA), and 1,6-diphenyl-1,3,5-hexatriene (DPH, CAS:1720-32-7, Cayman Chemical Company, Ann Arbor, MI, USA) were used. Polyethylene glycol 6000 (PEG, CAS:25322-68-3) was obtained from Fujifilm Wako Pure Chemical Corporation, Osaka, Japan.

### 3.2. Synthesis of Amphiphilic Polypeptides, SL12, SL16, SL4A4L4, and SA2L12A2

The amphiphilic polypeptides PSar_28_-*b*-(l-Leu-Aib)_6_ (S28L12) and PSar_27_-*b*-(l-Leu-Aib)_8_ (S27L16) were synthesized as reported previously [21,22,25,27]. The amphiphilic polypeptides PSar_24_-*b*-(l-Leu-Aib)_2_-(l-Ala-Aib)_2_-(l-Leu-Aib)_2_ (S24L4A4L4) and PSar_26_-*b*-(l-Ala-Aib)-(l-Leu-Aib)_6_-(l-Ala-Aib) (S26A2L12A2) were synthesized by modifying a reported synthesis scheme [21,22,24,27] (Schemes S1 and S2). Briefly, dipeptide units of Leu-Aib and Ala-Aib were synthesized with traditional condensation, and the 12 and 16 mer hydrophobic peptide was obtained by combining their units with sequential liquid-phase synthesis. Finally, polysarcosine was elongated from the *N*-terminal of their hydrophobic peptide with the ring-opening polymerization of sarcosine-*N*-carboxyanhydrides. All synthesized compounds were confirmed by ^1^H NMR spectroscopy and matrix-assisted laser desorption time-of-flight mass spectrometry (MALDI-TOF MS).

S24L4A4L4: ^1^H NMR (400 MHz, CD_3_OD) δ (ppm) 8.0–7.4 [m, 12H, amide], 4.4–3.9 [br, 54H, LeuC^α^*H*, AlaC^α^*H*, (SarC*H*_2_)_24_], 3.69 [s, 3H, OC*H*_3_], 3.3–2.8 [m, 72H, (Sar N-C*H*_3_)_24_], 2.1–1.4 [m, 63H, LeuC*H*_2_, LeuC^γ^*H*, AlaC*H*_3_, Aib(C*H*_3_)_2_, Boc(C*H*_3_)_3_], 1.1–0.8 [m, 24H, Leu(C*H*_3_)_2_]. MALDI-TOF MS calculated for C_129_H_222_N_36_O_39_Na: [M + Na]^+^ *m*/*z* 2922.639, found: 2922.919.

S26A2L12A2: ^1^H NMR (400 MHz, CD_3_OD) δ (ppm) 8.3–7.3 [m, 16H, amide], 4.6–3.8 [br, 60H, AlaC^α^*H*, LeuC^α^*H*, (SarC*H*2)26], 3.66 [s, 3H, OC*H*_3_], 3.3–2.8 [m, 78H, (Sar N-C*H*_3_)_26_], 2.0–1.3 [m, 81H, LeuC*H*_2_, LeuC^γ^*H*, Aib(C*H*_3_)_2_, Boc(C*H*_3_)_3_, AlaC*H*_3_], 1.1–0.8 [m, 36H, Leu(C*H*_3_)_2_]. MALDI-TOF MS calculated for C_155_H_268_N_42_O_45_Na: [M+Na]^+^ *m/z* 3460.997, found: 3460.913.

### 3.3. Preparation of Peptide Assemblies

For nanotube preparation, the ethanol injection method was used. Amphiphilic polypeptides (20 mg) were dissolved in ethanol (400 µL) to form stock solutions. An aliquot (10 µL) was then injected into milli-Q water (990 µL) during continued stirring at r.t. for 15 min. The dispersion was heated to 90 °C for 1–6 h and cooled to r.t.

For the double-layer nanotubes, PEG 6,000 (150 µL) was added to SL12 nanotube dispersion (350 µL) (final conc. of PEG 30%), and then the mixture was stirred at r.t. for 10 min. Subsequently, SA2L12A2 stock solution (3.5 µL) was injected into the dispersion with stirring, which was continued for 20 min. The mixture was then heated to 90 °C for 3 h, cooled to r.t., and finally, the PEG content was removed by dialysis (dialysis membrane: 10 K) over 2 days.

### 3.4. Transmission Electron Microscopy (TEM)

TEM images were acquired using a JEOL JEM-1230 at an accelerating voltage of 80 kV. For observations, a drop of dispersion was mounted on a carbon-coated Cu grid (Okenshoji Co., Ltd., Japan) and negatively stained with 2% samarium acetate, followed by removal of the excess liquid with a filter paper.

### 3.5. Circular Dichroism (CD)

CD measurements were carried out on a JASCO J-720 (JEOL, Tokyo, Japan) using a cell with an optical path length of 1 cm. Data were recorded at 25 °C. Peptide solution (0.15 mM) was used for self-assembly in milli-Q water, and the dispersion was diluted ten-fold before CD data collection.

### 3.6. Membrane Fluidity by DPH

The membrane fluidities of the peptide assemblies were ascertained at room temperature using 1,6-diphenyl-1,3,5-hexatriene (DPH) as a polarization agent (JASCO FP-6500) [40,41,42]. In each trial, a 250 µM ethanol solution of DPH (4 µL) was added to a 0.15 mM (0.5 mg/mL) assembly dispersion, after which each sample was incubated for 60 min in the dark. The excitation and emission wavelengths were 360 and 430 nm, respectively, and the excitation was vertically polarized while the emission was recorded in both parallel *I*_∥_ (0°, 0°) and perpendicular *I*_⊥_ (0°, 90°) modes. The polarization (*P*) of the DPH was calculated using the formula *P* = (*I*_∥_ − *GI*_⊥_)/(*I*_∥_ + *GI*_⊥_), where *G* is a correction factor equal to *i*_⊥_/*i*_∥_ and *i*_⊥_ and *i*_∥_ are the perpendicular (90°, 0°) and parallel (90°, 90°) emission intensities, respectively, using horizontally polarized light.

### 3.7. Membrane Fluidity by Laurdan

The membrane fluidities of the peptide assemblies were measured with *N*, *N*-dimethyl-6-dodecanoyl-2-naphthylamine (Laurdan). Laurdan is a fluorescent dye that is sensitive to the polarity of its surrounding environment [35,36,43,44]. A general polarization value, *GP*_340_, was calculated as *GP*_340_ = (*I*_440_ − *I*_490_) / (*I*_440_ + *I*_490_), where *I*_440_ and *I*_490_ are the emission intensities at 440 and 490 nm of Laurdan excited at 340 nm.

## 4. Conclusions

To investigate the effect of the Leu position in the helix on the helical packing and the morphology of the assembly directed by the packing, SL4A4L4, SL12, SA2L12A2, and SL16 were synthesized. SL4A4L4 and SL12 formed isotropic spheres and anisotropic tubes, respectively. It was determined that the LxxxLxxxL sequence is an important factor for tube-forming packing. In fact, SA2L12A2 with an L12 sequence in the middle section of the helix, self-assembled into nanotubes and the Ala-Aib part, did not disrupt the packing formation. In addition, it was shown that the Leu-Aib positioned at the *N*- and *C*-terminal of L16 strongly affect the helix packing because SL16 showed only vesicle formation. The concave–convex interaction induced by the Leu side chain is a promising tool for precisely controlling the assembly morphology. The A2L12A2 sequence made the peptide membrane slightly more flexible and planer than L12, as indicated by the 120 nm diameter nanotube of SA2L12A2 compared with the 80 nm diameter nanotube of SL12. By self-assembling SA2L12A2 in the SL12 nanotube dispersion, double-layer nanotubes with uniform diameters for the inner and outer nanotubes composed of SL12 and SA2L12A2 were obtained.

## Data Availability

Not applicable.

## Supplementary Materials

The following are available online at https://www.mdpi.com/article/10.3390/ijms222112075/s1.
