# Supplementary material for: Tubular Assembly Formation Induced by Leucine Alignment along the Hydrophobic Helix of Amphiphilic Polypeptides
Source: Int J Mol Sci. 2021 Nov 8;22(21):12075. doi: 10.3390/ijms222112075 (PMC8584449; doi:10.3390/ijms222112075)
Supplement: Supplementary file 1 [file ijms-22-12075-s001.zip › ijms-1417112-supplementary.pdf]

Supporting Information

Tubular Assembly Formation Induced by Leucine Alignment along Hydrophobic Helix of Amphiphilic Polypeptide

Mohammed A. Abosheasha[1,2], Toru Itagaki[1], Yoshihiro Ito[1,2,3], Motoki Ueda[1,3]*

[1]RIKEN Cluster for Pioneering Research (CPR), 2-1 Hirosawa, Wako, Saitama 351-0198, Japan, [2]Department of Biological Sciences, Graduate School of Science, Tokyo Metropolitan University, 1-1 Minami-Osawa, Hachioji, Tokyo 192-0397, Japan, [3]RIKEN Center for Emergent Matter Science (CEMS), 2-1 Hirosawa, Wako, Saitama 351-0198, Japan.

CONTENTS

SI-1. Synthesis of amphiphilic polypeptides (Scheme S1–S2 and Fig. S1–S2)

SI-2. ^{1}H NMR and MS spectra of SL12 and SL16 (Fig. S3 and S4)

SI-3. TEM images of DLPANT prepared by self-assembly of SA2L12A2 in SL12 nanotube dispersion (Fig. S5).

SI-4. TEM images of SA2L12A2 assembly in SL12 nanotube dispersion at 50 or 70 °C (Fig. S6)

SI-5. TEM images of DLPANT prepared by self-assembly of SA2L12A2 in SL12 nanotube dispersion with PEG (Fig. S7).

SI-1. Synthesis of amphiphilic polypeptides (Scheme S1–S2 and Fig. S1–S2)

Synthesis of SA2L12A2

SA2L12A2 was synthesized according to the scheme S1.

Scheme S1. Synthesis of SA2L12A2.

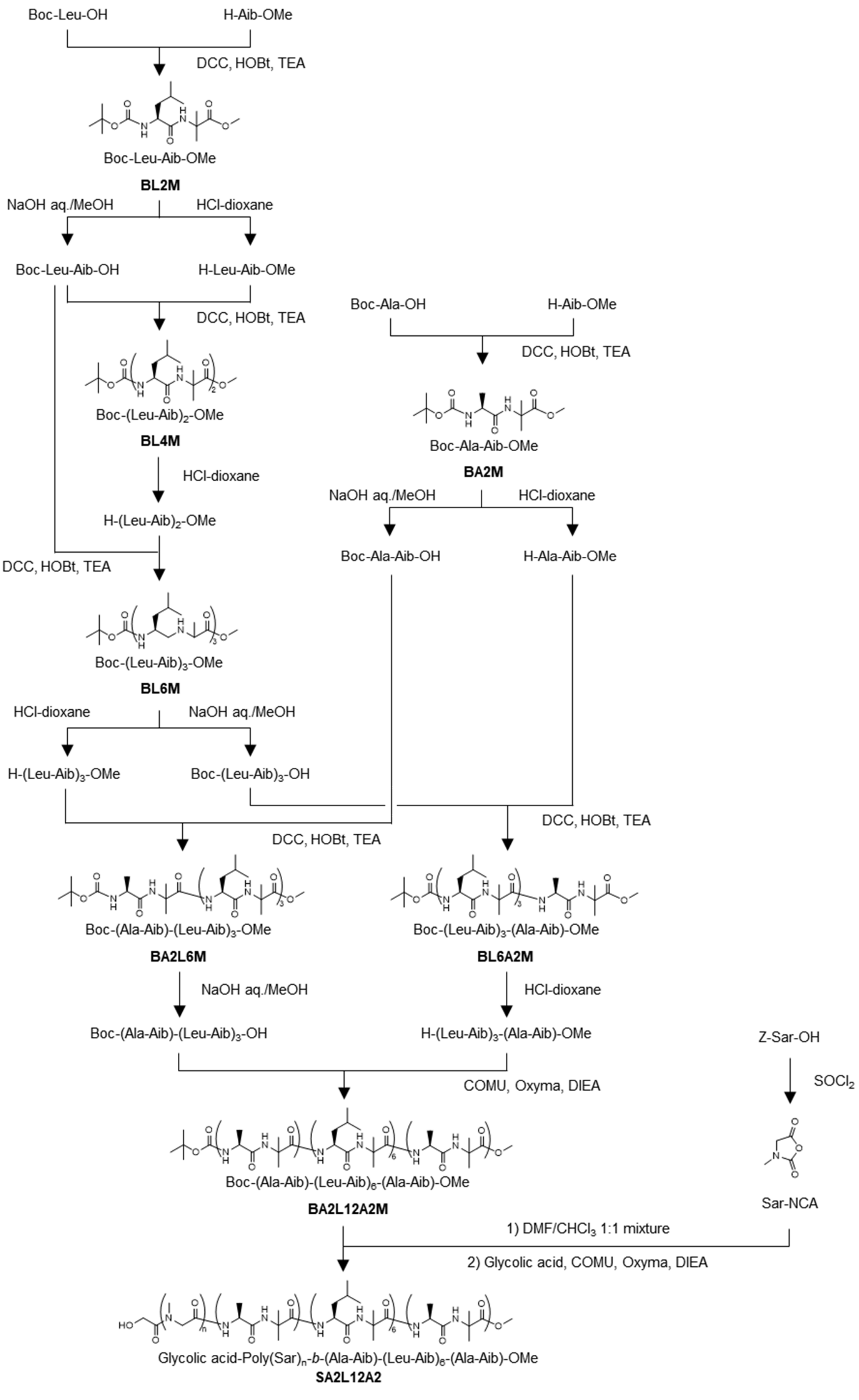

BA2L6M

To a solution of HL6M (580 mg, 925.28 μmol) and BA2OH (310.3 mg, 1.13 mmol) in super dehydrated DMF (4 ml), a solution of N,N′-Dicyclohexylcarbodiimide (DCC) (290.2 mg, 1.41 mmol), Hydroxybenzotriazole (HOBt) (192.5 mg, 1.42 mmol) and Triethylamine (TEA) (285 μl, 2.04 mmol) in super dehydrated DMF (4 ml) was added at 0 °C. The reaction mixture was stirred at 0 °C for 0.5 h under argon atmosphere and then at room temperature for 18 h. The reaction was monitored by thin layer chromatography (TLC) with ninhydrin staining. The reaction mixture was concentrated by rotary evaporation and dried under vacuum. The dried crude was dissolved in chloroform and then was washed three times with saturated NaHCO3 aq., three times with 4% $KHSO_4$ aq., then once with sat. NaCl aq. and dried over $MgSO_4$. The crude product was purified by column chromatography (silica gel, eluent: chloroform/methanol = 50/1). The fractions were monitored by thin layer chromatography (TLC) with ninhydrin staining. The product was obtained as a white solid (520 mg, 64%).

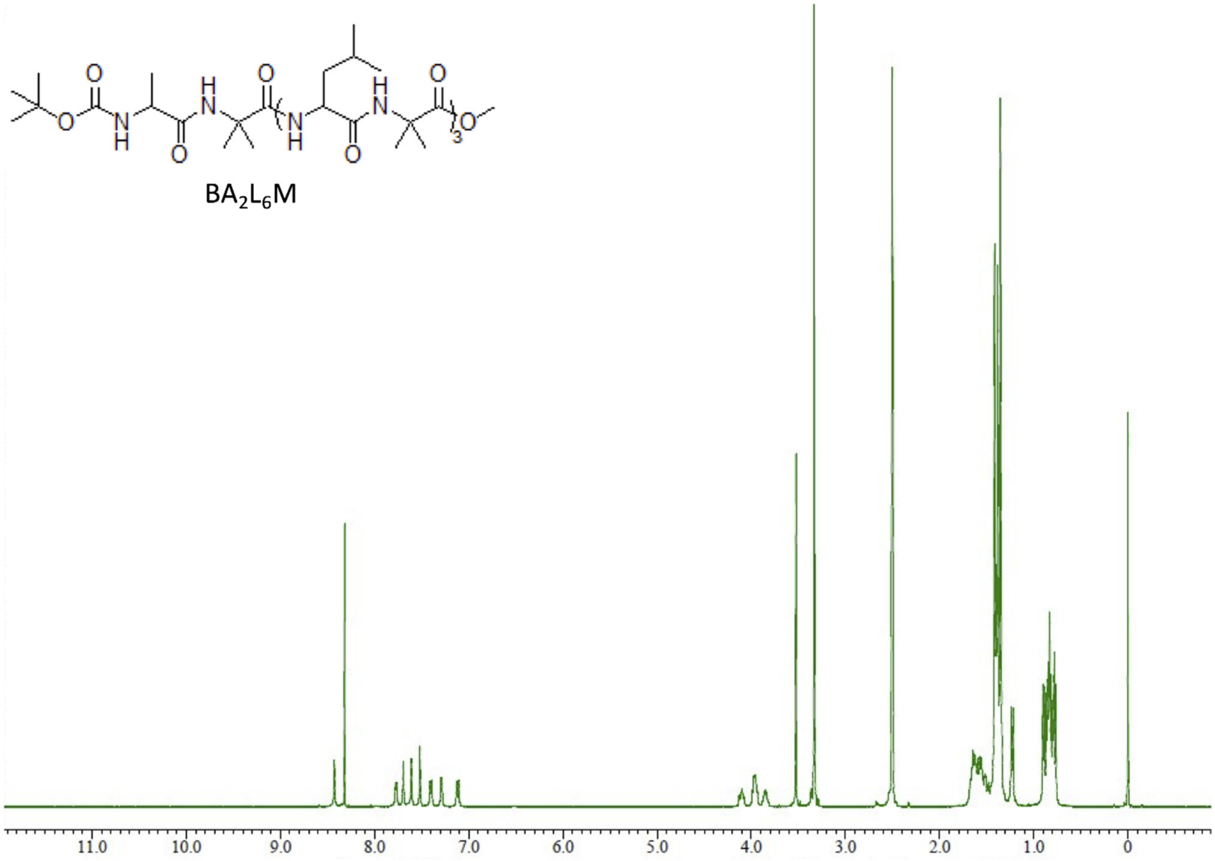

^{1}H NMR spectra of BA2L6M. ^{1}H NMR (400 MHz, $(CD_3)_2SO$) δ (ppm) 8.3–7.0 [m,8H, amide], 4.6–3.8 [br, 4H, LeuC$^{\alpha}$*H*], 3.66 [s, 3H, OC*H*$_3$], 2.0–1.3 [m, 45H, LeuC*H*$_2$, LeuC$^{\gamma}$*H*, Aib(C*H*$_3$)$_2$, Boc(C*H*$_3$)$_3$, AlaC*H*$_3$], 1.1–0.8 [m, 18H, Leu(C*H*$_3$)$_2$].

MALDI-TOF MS calculated for $C_{43}H_{78}N_8O_{11}Na^+$ $[M+Na]^+$ *m/z* 905.579, found: 905.574.

BL6A2M

To a solution of BL6OH (460 mg, 645.22 µmol) and HA2M (169.4 mg, 897.85 µmol) in super dehydrated DMF (4 ml), a solution of DCC (211.9 mg, 1.03 mmol), HOBt (137.3 mg, 1.02 mmol) and TEA (200 µl, 1.43 mmol) in super dehydrated DMF (4 ml) was added at 0 °C. The reaction mixture was stirred at 0 °C for 0.5 h under argon atmosphere and then at room temperature for 24 h. The reaction was monitored by TLC with ninhydrin staining. The reaction mixture was concentrated by rotary evaporation and dried under vacuum. The dried crude was dissolved in chloroform and then was washed four times with saturated $NaHCO_3$ aq., three times with 4% $KHSO_4$ aq., then once with sat. NaCl aq. and dried over $MgSO_4$. The crude product was purified by column chromatography (silica gel, eluent: chloroform/methanol = 50/1). The fractions were monitored by TLC with ninhydrin staining. The product was obtained as a white solid (360 mg, 63.2%).

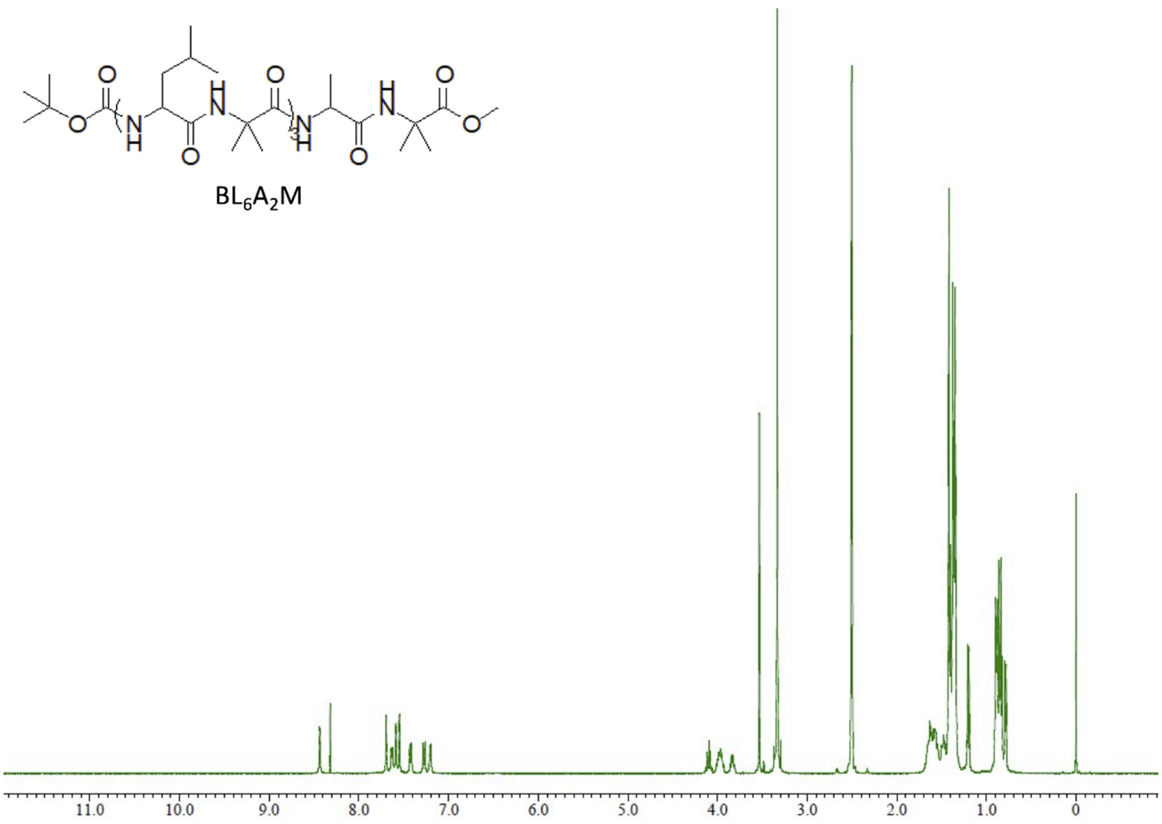

^{1}H NMR spectra of BL6A2M. ^{1}H NMR (400 MHz, $(CD_3)_2SO$) δ (ppm) 8.3–7.0 [m,8H, amide], 4.6–3.8 [br, 4H, LeuC$^{\alpha}$*H*], 3.66 [s, 3H, OCH_3], 2.0–1.3 [m, 45H, LeuCH_2, LeuC$^{\gamma}$*H*, Aib(CH_3)$_2$, Boc(CH_3)$_3$, AlaCH_3], 1.1–0.8 [m, 18H, Leu(CH_3)$_2$].

MALDI-TOF MS calculated for $C_{43}H_{78}N_8O_{11}Na^+$ $[M+Na]^+$ *m/z* 905.579, found: 905.572.

BA2L12A2M

After methyl ester deprotection of BA2L6M by using NaOH aq./Methanol, BA2L6OH was obtained after neutralization of reaction mixture by 1N HCl then washing once by 4% $KHSO_4$ aq. In addition, *tert*-butyloxycarbonyl (Boc) group deprotection of BL6A2M was performed by using 4N HCl dioxane. The HL6A2M was obtained after concentration by rotary evaporation and drying under vacuum. Then the condensation reaction was achieved as the following: to a solution of BA2L6OH (79.6 mg, 91.59 μmol) and HL6A2M (86.06 mg, 109.9 μmol) in super dehydrated DMF (3 ml), a solution of COMU (58.84 mg, 137.38 μmol), ethyl cyanohydroxyiminoacetate (Oxyma) (19.52 mg, 137.38 μmol) in super dehydrated DMF (2 ml) was added at 0 °C then N, N-Diisopropylethylamine (DIEA) (40 μl, 201.49 μmol) was added. The reaction mixture was stirred at 0 °C for 0.5 h under argon atmosphere and then at room temperature for 24 h. The reaction was monitored by TLC with ninhydrin staining. The reaction mixture was concentrated by rotary evaporation and dried under vacuum. The dried crude was dissolved in chloroform and then was washed three times with saturated $NaHCO_3$ aq., three times with 4% $KHSO_4$ aq., then once with sat. NaCl aq. and dried over $MgSO_4$. The crude product was purified by column chromatography (silica gel, eluent: chloroform/methanol = 10/1). The fractions were monitored by TLC with ninhydrin staining. The product was purified using Sephadex LH-20 column, with MeOH: CH3Cl = 1:1 as the eluent. Fractions were

identified using UV spectrum at 280 nm. The product was obtained as a white solid (104 mg, 70%).

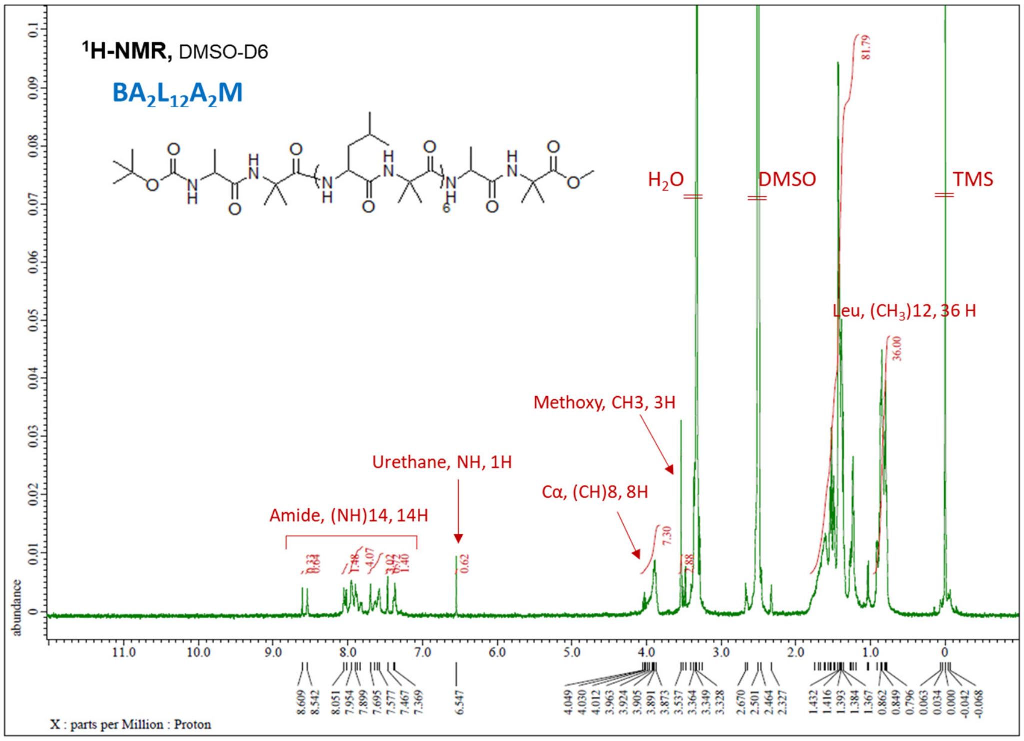

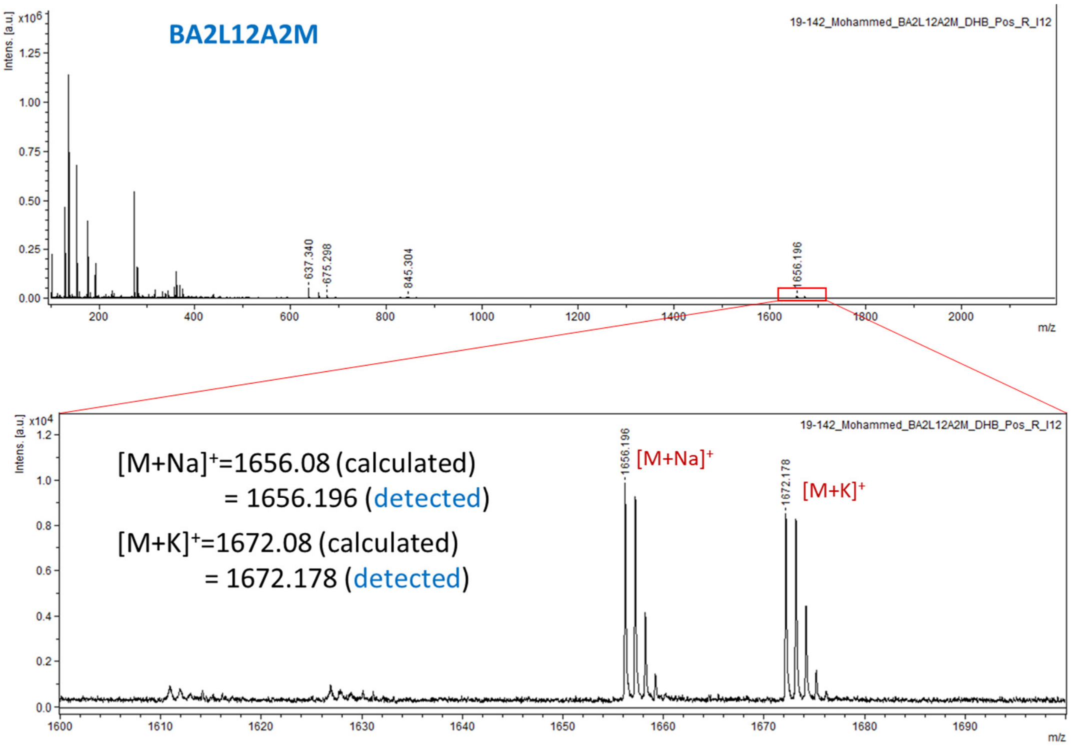

^{1}H NMR spectra of BA2L12A2M. ^{1}H NMR (400 MHz, $(CD_3)_2SO$) δ (ppm) 8.6–7.3 [m,14H,

amide], 6.5 [s, 1H, urethane] 4.1–3.8 [br, 8H, AlaC$^{\alpha}$*H*, LeuC$^{\alpha}$*H*], 3.66 [s, 3H, OCH_3], 1.8–1.2 [m, 81H, LeuCH_2, LeuC$^{\gamma}$*H*, Aib(CH_3)$_2$, Boc(CH_3)$_3$, AlaCH_3], 1.1–0.8 [m, 36H, Leu(CH_3)$_2$].

MALDI-TOF MS, calculated $[M+Na]^+$ *m/z* 1656.08, found: 1656.196.

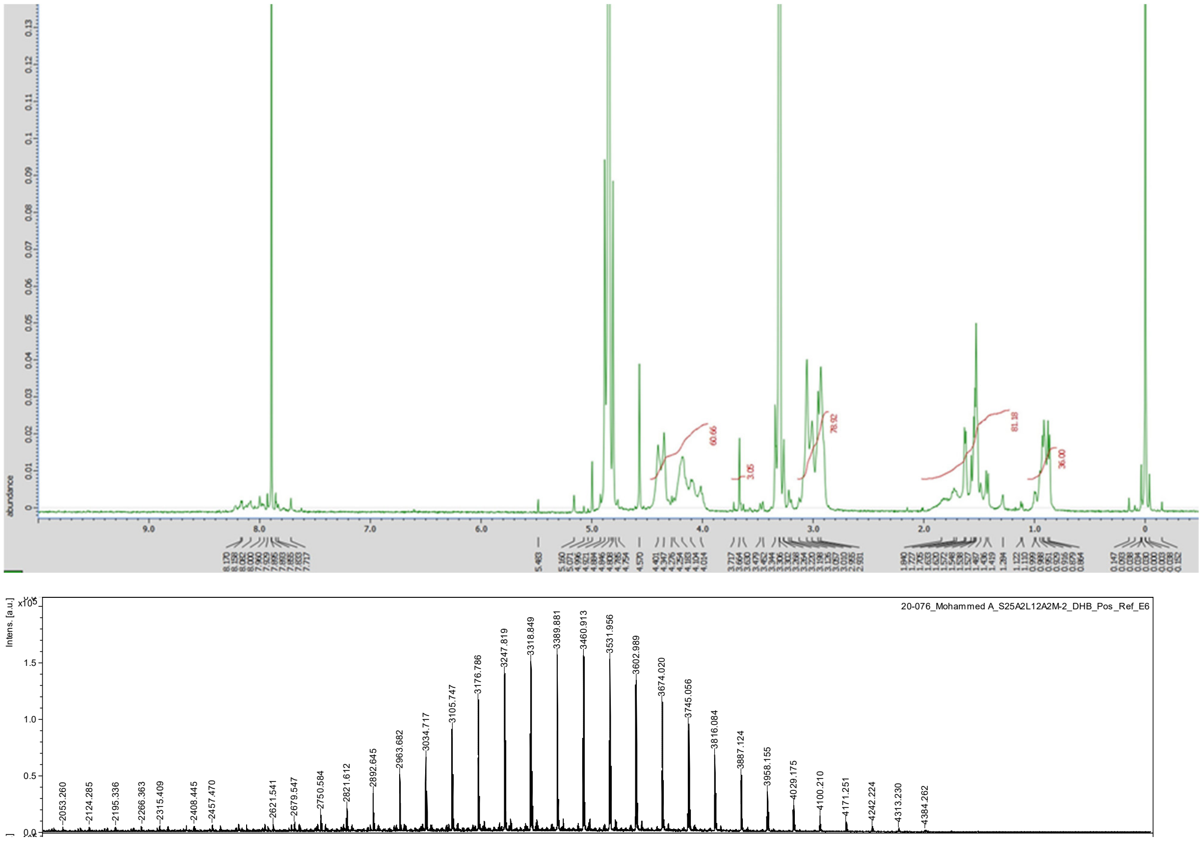

Fig. S1. ^{1}H NMR spectra and MALDI-TOF MS spectra of SA2L12A2.

<u>SA2L12A2</u>

^{1}H NMR (400 MHz, CD_3OD) δ (ppm) 8.3–7.8 [m, 16H, amide], 4.6–3.8 [br, 60H, LeuC$^{\alpha}$*H*, AlaC$^{\alpha}$*H*, (SarC*H*$_2$)$_{26}$], 3.66 [s, 3H, OC*H*$_3$], 3.1–2.9 [m, 78H, (SarC*H*$_3$)$_{26}$], 2.0-1.2 [m, 81H, LeuC*H*$_2$, LeuC$^{\gamma}$*H*, Aib(C*H*$_3$)$_2$, Boc(C*H*$_3$)$_3$, AlaC*H*$_3$], 1.1–0.8 [m, 36H, Leu(C*H*$_3$)$_2$].

MALDI-TOF MS calculated for $C_{155}H_{268}N_{42}O_{45}Na^+$ $[M+Na]^+$ 3461, found: 3460.913.

Synthesis of SL4A4L4

SL4A4L4 was synthesized according to the below scheme S2.

Scheme S2. Synthesis of SL4A4L4.

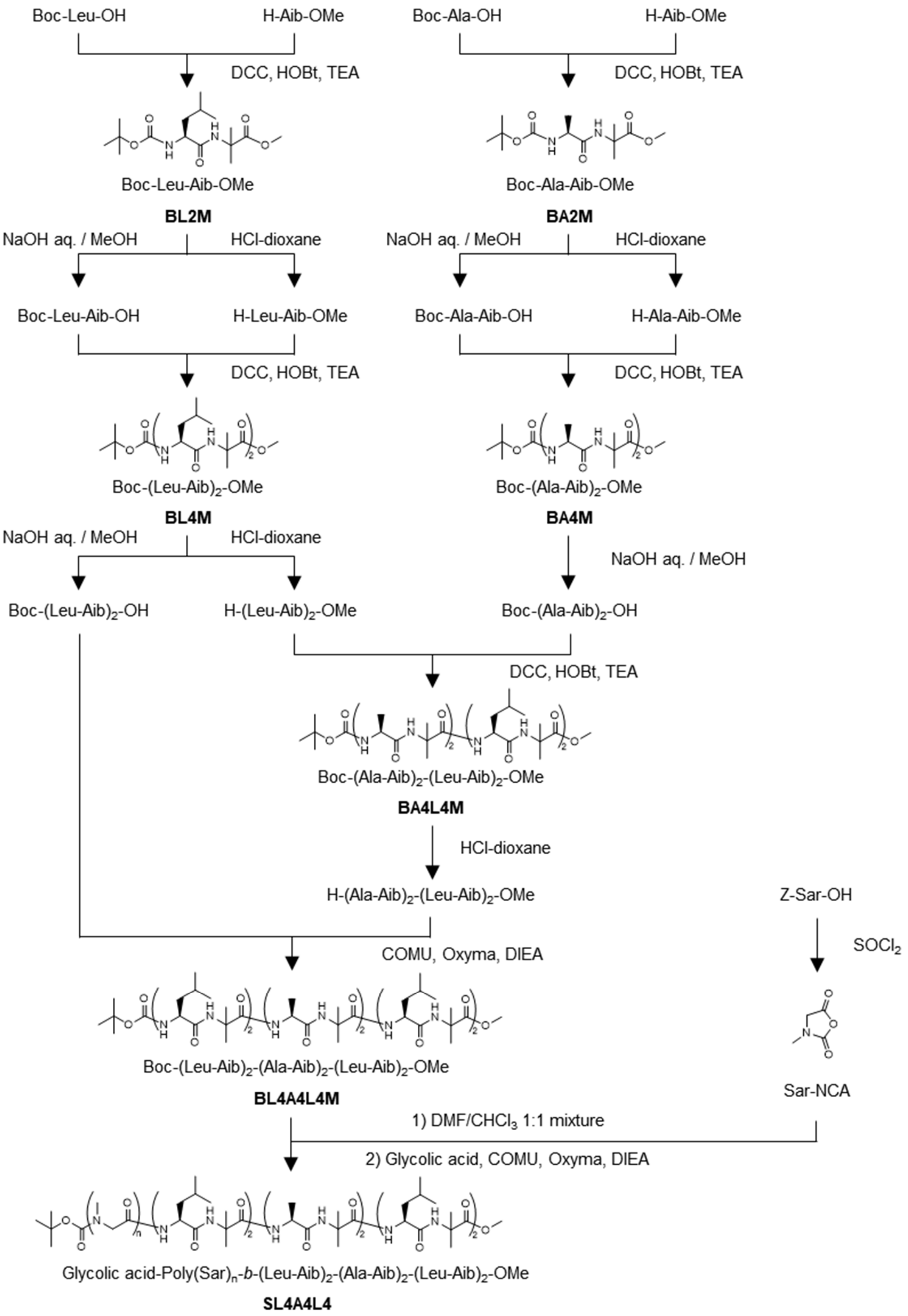

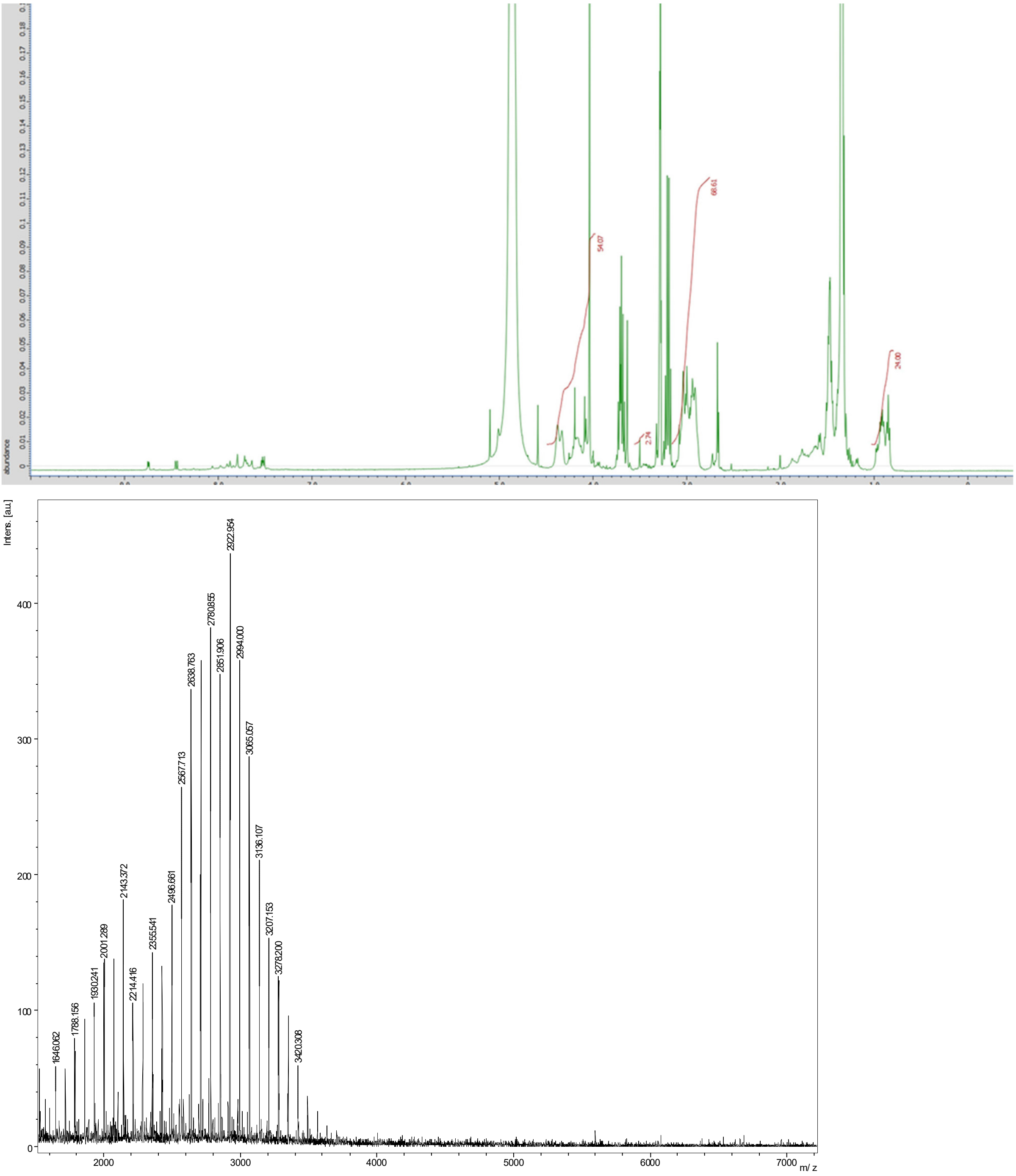

Fig. S2. ^{1}H NMR spectra and MALDI-TOF MS spectra of SL4A4L4.

<u>SL4A4L4</u>

^{1}H NMR (400 MHz, CD_3OD) δ (ppm) 8.3–7.8 [m, 12H, amide], 4.6–3.8 [br, 54H, LeuC$^{\alpha}$*H*, AlaC$^{\alpha}$*H*, (SarC*H*$_2$)$_{24}$], 3.66 [s, 3H, OC*H*$_3$], 3.1–2.9 [m, 72H, (SarC*H*$_3$)$_{24}$], 2.0-1.2 [m, 63H,

LeuCH_2, Leu$C^{\gamma}H$, Aib$(CH_3)_2$, Boc$(CH_3)_3$, AlaCH_3], 1.1–0.8 [m, 24H, Leu$(CH_3)_2$].

MALDI-TOF MS calculated for $C_{129}H_{222}N_{36}O_{39}Na^+$ $[M+Na]^+$ *m/z* 2922.649, found: 2922.954

SI-2. ^{1}H NMR and MS spectra of SL12 and SL16 (Fig. S3 and S4)

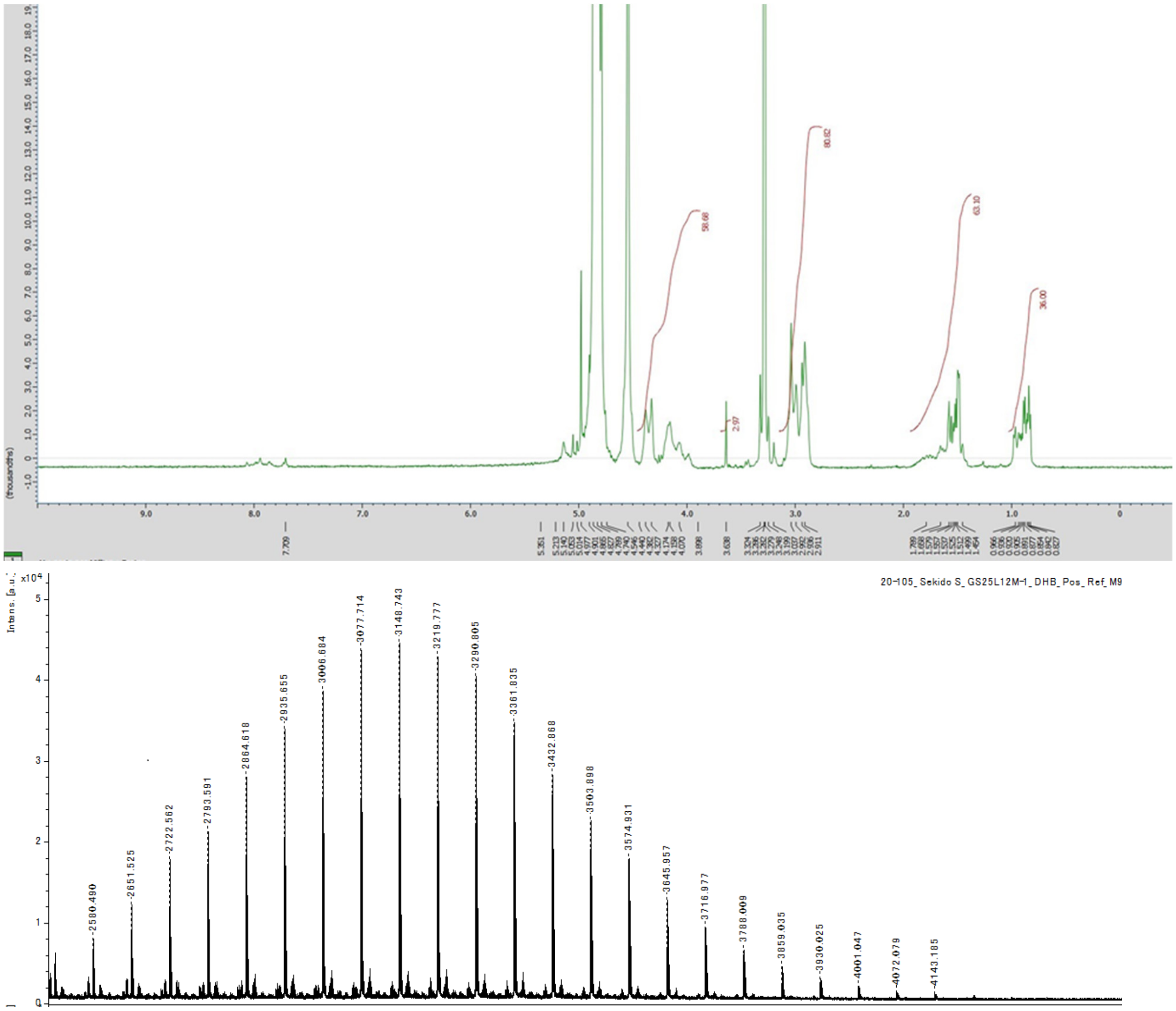

Fig. S3. 1H NMR spectra and MALDI-TOF MS spectra of SL12

<u>SL12</u>

1H NMR (400 MHz, CD_3OD) δ (ppm) 8.0–7.4 [m, 12H, amide], 4.4–3.9 [br, 60H, LeuC$^{\alpha}$*H*, (SarC*H*$_2$)$_{27}$], 3.66 [s, 3H, OC*H*$_3$], 3.3–2.8 [m, 81H, (SarC*H*$_3$)$_{27}$], 2.0–1.4 [m, 63H, LeuC*H*$_2$, LeuC$^{\gamma}$*H*, Aib(C*H*$_3$)$_2$, Boc(C*H*$_3$)$_3$], 1.1–0.8 [m, 36H, Leu(C*H*$_3$)$_2$].

MALDI-TOF MS calculated $C_{147}H_{254}N_{40}O_{43}Na$ for $[M+Na]^+$ *m/z* 3290.882, found: 3290.805.

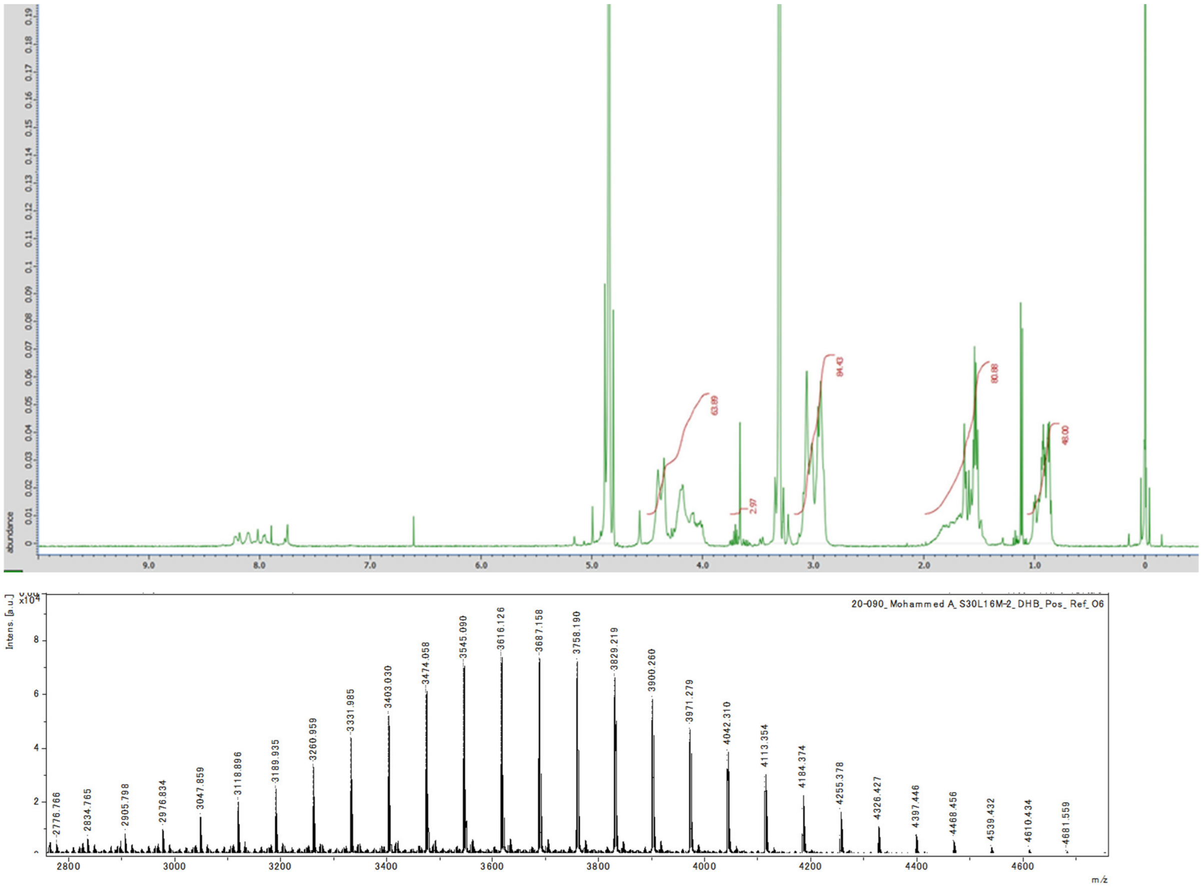

Fig. S4. 1H NMR spectra and MALDI-TOF MS spectra of SL16

<u>SL16</u>

1H NMR (400 MHz, CD_3OD) δ (ppm) 8.0–7.4 [m, 17H, amide], 6.6 [s, 1H, urethane], 4.5–3.9 [br, 64H, LeuC$^{\alpha}$*H*, (SarC*H*$_2$)$_{28}$], 3.66 [s, 3H, OC*H*$_3$], 3.3–2.8 [m, 84H, (SarC*H*$_3$)$_{28}$], 2.0–1.4 [m, 81H, LeuC*H*$_2$, LeuC$^{\gamma}$*H*, Aib(C*H*$_3$)$_2$, Boc(C*H*$_3$)$_3$], 1.1–0.8 [m, 48H, Leu(C*H*$_3$)$_2$].

MALDI-TOF MS calculated $C_{167}H_{290}N_{44}O_{47}Na^+$ $[M+Na]^+$ *m/z* 3687.165, found: 3687.158.

SI-3. TEM images of DLPANT prepared by self-assembly of SA2L12A2 in SL12 nanotube dispersion (Fig. S5).

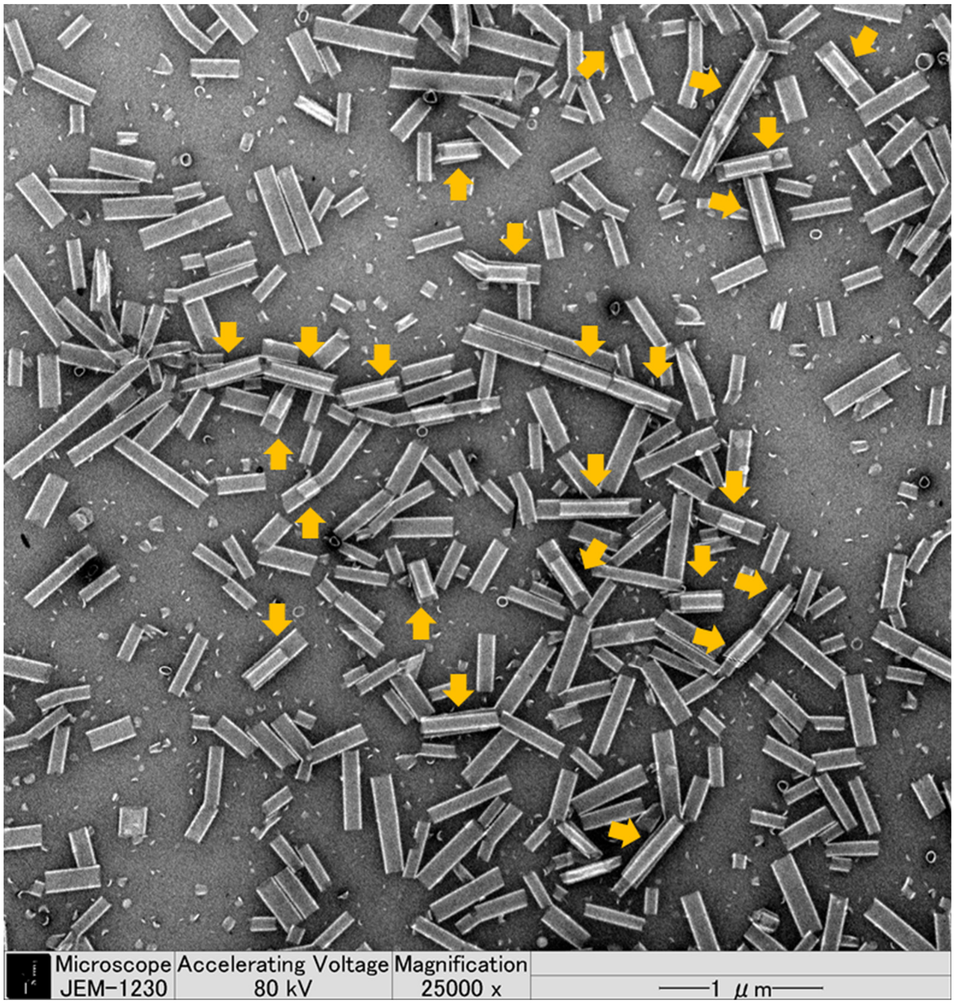

Fig. S5. TEM image of DLPANT prepared by self-assembly of SA2L12A2 in SL12 nanotube dispersion after heat treatment at 90 °C for 6 h. The yellow arrow means double layer nanotube.

SI-4. TEM images of SA2L12A2 assembly in SL12 nanotube dispersion at 50 or 70 °C (Fig. S6).

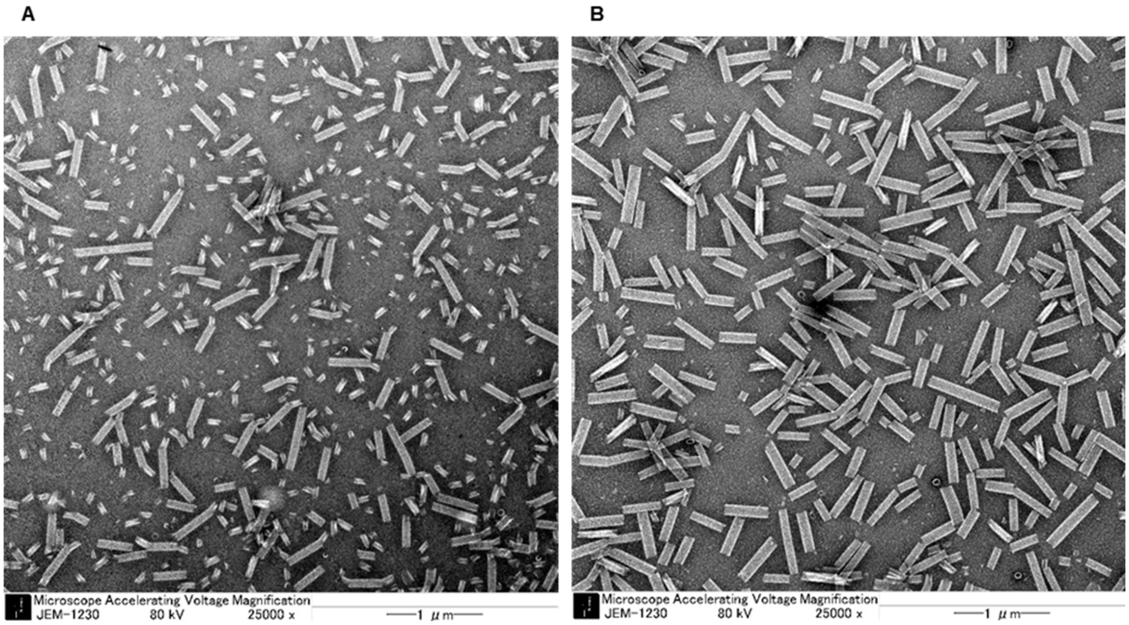

Fig. S6. TEM images of assembly prepared by self-assembly of SA2L12A2 in SL12 nanotube dispersion after heat treatment at 50 °C (A) and 70 °C (B) for 6 h.

SI-5. TEM images of DLPANT prepared by self-assembly of SA2L12A2 in SL12 nanotube dispersion with PEG (Fig. S7).

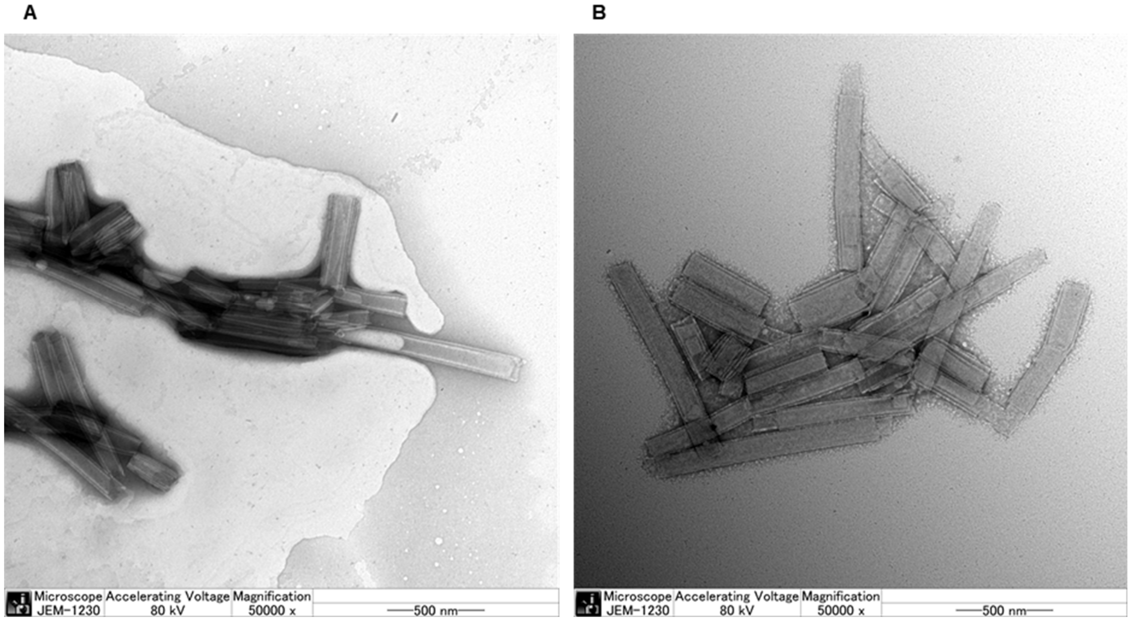

Fig. S7. TEM images of DLPANT and MLPANT prepared by self-assembly of SA2L12A2 in SL12 nanotube with 30% PEG before (A) and after dialysis (B).
